# Combination of toothbrushing and chlorhexidine compared with exclusive use of chlorhexidine to reduce the risk of ventilator-associated pneumonia: A systematic review with meta-analysis

**DOI:** 10.6061/clinics/2021/e2659

**Published:** 2021-05-27

**Authors:** Pedro Urquiza Jayme Silva, Luiz Renato Paranhos, Daniela Meneses-Santos, Cauane Blumenberg, Dhiancarlo Rocha Macedo, Sérgio Vitorino Cardoso

**Affiliations:** I Programa de Pos-Graduacao em Odontologia, Faculdade de Odontologia, Universidade Federal de Uberlandia, Uberlandia, MG, BR.; II Area de Odontologia Preventiva e Social, Faculdade de Odontologia, Universidade Federal de Uberlandia, Uberlandia, MG, BR.; III Programa de Residencia em Cirurgia e Traumatologia Buco-Maxilo-Facial, Faculdade de Medicina, Universidade Federal de Uberlandia, Uberlandia, MG, BR.; IV Programa de Pos-Graduacao em Epidemiologia, Faculdade de Medicina, Universidade Federal de Pelotas, Pelotas, RS, BR.; V Hospital Odontologico, Universidade Federal de Uberlandia, Uberlandia, MG, BR.; VI Area de Patologia, Faculdade de Odontologia, Universidade Federal de Uberlandia, Uberlandia, MG, BR.

**Keywords:** Chlorhexidine, COVID-19, Tooth Brushing, Ventilator-Associated Pneumonia

## Abstract

This study aimed to compare the effectiveness of 0.12% chlorhexidine alone and 0.12% chlorhexidine in combination with toothbrushing to prevent ventilator-associated pneumonia (VAP) in mechanically ventilated patients.

The Embase, Latin American and Caribbean Health Science Literature, PubMed, Scientific Electronic Library Online, Scopus, LIVIVO, Web of Science, Cochrane Library, OpenThesis, and Open Access Thesis and Dissertations databases were used. Only randomized controlled trials without restrictions on the year or language of publication were included. Two reviewers assessed the risk of bias using the Joanna Briggs Institute Critical Appraisal Tool. A meta-analysis using a random-effects model estimated the combined relative risk (RR). The Grading of Recommendations, Assessment, Development and Evaluations approach was used to assess the certainty of the evidence.

Initially, 2,337 studies were identified, of which 4 were considered in the systematic review and 3 in the meta-analysis (total sample: 796 patients). The studies were published between 2009 and 2017. All eligible studies had a low risk of bias. The meta-analysis revealed that the risk of VAP was 24% lower in patients receiving chlorhexidine combined with toothbrushing than in those receiving chlorhexidine alone (RR: 0.76; 95% confidence interval: 0.55-1.06), with moderate certainty of evidence and without statistical significance.

In conclusion, considering the limitations of this study, a standard protocol for the prevention of VAP is not yet recommended. More studies with larger sample sizes are needed to draw strong conclusions. However, considering that toothbrushing is a simple intervention, it should be a common practice in mechanically ventilated patients, especially among patients with coronavirus disease.

## INTRODUCTION

Ventilator-associated pneumonia (VAP) is defined as pneumonia occurring more than 48h after the onset of mechanical ventilation (1). It affects 10%-20% of patients who receive mechanical ventilation for more than 48h. VAP is diagnosed based on the following criteria: presence of purulent sputum, fever (>38°C) or hypothermia (<35.5°C), leukocytosis (>10,000 mm^3^) or leukopenia (<4,000 mm^3^), positive bacterial culture of respiratory secretions (>10^6^ cfu/mL), and radiography showing additional or progressive pulmonary infiltrates (2).

Several risk factors are associated with VAP, such as older age, male sex, increased time on mechanical ventilation, sedation, heart and lung disease, regurgitation, aspiration, prior antibiotic therapy, and invasive operations (3). Burns are also a risk factor of VAP through pulmonary inflammation resulting from direct lung injury or systemic immune dysfunction (4). Genetic polymorphisms related to inflammatory mediators may also increase the risk of developing VAP, possibly because of an ineffective response to bacteria (5).

Currently, the global pandemic against severe acute respiratory syndrome coronavirus 2 (SARS-CoV-2) has resulted in a higher frequency of patients requiring invasive mechanical ventilation (6). Likewise, prone positioning, heavy sedation, and treatment with neuromuscular blockers, in addition to clear evidence of prolonged immunosuppression, including deep lymphopenia, represent a risk for acquiring secondary infections, including VAP (6,7). VAP is a complication in patients hospitalized for coronavirus disease (COVID-19) (8-10).

Oral hygiene using a variety of procedures is an important measure to prevent VAP (11). For instance, aspiration of secretions, toothbrushing, or dental and mucosal cleansing with chlorhexidine (CHX) may reduce the risk of VAP (12). CHX is a cationic biguanide that binds to the bacterial cell walls, thus impairing and even perforating phospholipid membranes (13,14). The effect may be bacteriostatic or bactericidal depending on the concentration of the product (15). Its use for oral hygiene in patients under mechanical ventilation reduces the risk of VAP (16-19). As a mouthwash, CHX reduces bacterial colonization in the oral cavity (20,21). However, the presence of a biofilm on the surface of the teeth limits the action of any mouthwash (22). Thus, prior mechanical disruption of dental biofilms through toothbrushing improves the effect of CHX (23-25) and, hence, prevents VAP (25-27).

High CHX concentrations have been associated with adverse effects (28). Dental discoloration and oral mucosa irritation were attributed to the use of 0.2% and 2% CHX (29). Lesions in the oral mucosa, such as erosive lesions, ulcerations, white/yellow plaque formation, and mucosal bleeding, have been observed in patients admitted in the intensive care units (30). By contrast, when 0.12% CHX was applied, it was effective in preventing VAP in surgical patients (31).

This study aimed to compare the reduction in the risk of VAP between the use of oral 0.12% CHX combined with toothbrushing and use of 0.12% CHX alone in the prevention of VAP through systematic review and meta-analysis.

## METHODS

### Protocol and registration

This systematic review adhered to the Preferred Reporting Items for Systematic Reviews and Meta-Analyses (PRISMA) recommendations (32) and Cochrane guidelines (33). It was recorded in the International Prospective Register of Systematic Reviews (PROSPERO) (CRD42020168844) (https://www.crd.york.ac.uk/PROSPERO/).

### Study design and eligibility criteria

This systematic review with meta-analysis was conducted based on the patient, intervention, comparison, outcome strategy and aimed at answering the following review question: “Is toothbrushing combined with the use of 0.12% CHX (intervention) more effective in preventing VAP (outcome) among patients under mechanical ventilation (population) than using CHX alone (comparison)?”

Randomized controlled trials that compared oral hygiene using 0.12% CHX with or without toothbrushing in adult patients (aged >18 years) under invasive (tracheal) mechanical ventilation were included in the study. There were no restrictions on the year, language, or publication status (published, accepted/ahead of print articles). Studies not related to the objective of the present study, non-original works (review articles, editorials, and books/book chapters), or papers with insufficient data (letters, personal opinions, and conference abstracts) were excluded.

### Sources of information and search

**Figure 1:**
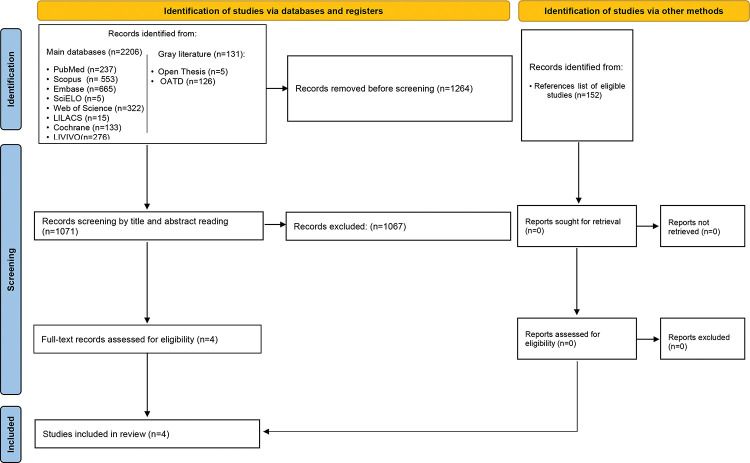
Flow chart of the study selection process.

The primary sources of studies were PubMed (including MedLine), Scopus, Embase, Scientific Electronic Library Online, Web of Science, Latin-American and Caribbean Health Sciences Literature, Cochrane, and LIVIVO databases. The Open Access Thesis and Dissertations and OpenGrey databases allowed access to the “gray literature” to avoid bias regarding the lack of published negative results (Figure 1). The sources of search descriptors were the Medical Subject Headings, Health Sciences Descriptors, and Emtree. Several combinations of the Boolean operators “AND” and “OR” enhanced the search strategy, as detailed in Table 1. The search terms were adapted to each database. Bibliographic research was performed until November 2019. The results obtained were imported to the software EndNote Web™ (Thomson Reuters, Toronto, Canada) and then into Microsoft Word™ 2010 (Microsoft™ Ltd., Washington, USA) for the automatic and conventional removal of duplicates, respectively.

### Study selection

Two independent reviewers (PUJS and DMS) previously calibrated 20% of the studies and reached an acceptable inter-examiner agreement (kappa>0.81). Then, these reviewers independently performed the eligibility review, with disagreements resolved by discussion with a third reviewer (LRP) until consensus was reached.

The study selection was performed in two stages. First, the analysis of the titles and abstracts (when available) led to the exclusion of articles not related to the topic of the present review. The second stage involved the evaluation of the full text of the remaining studies to verify their adherence to the eligibility criteria. In both stages, the reviewers had access to the names of the authors and journals. A thorough verification of the references of the eligible articles was performed to identify studies overlooked in the initial search. The excluded studies were registered separately, along with the reasons for exclusion. If any article could not be recovered, other study centers were contacted to retrieve the articles in their libraries. In the case of studies published in languages other than English or Portuguese, the full text was translated.

### Data collection

Two reviewers (PUJS and DMS) examined the selected papers to collect the following information: identification (author, year, and country of the research), sample features (number of patients, sex distribution, mean age, and Acute Physiology and Chronic Health Evaluation score) (34,35), and the main results (ventilation time, microbiota assessment, VAP incidence, mortality, and conclusions). The corresponding authors were contacted by email (up to three times over two weeks) to obtain relevant information on missing or unclear data.

To ensure consistency, the reviewers (PUJS and DMS) extracted the information jointly from an eligible study. These reviewers discussed to resolve initial discrepancies, and a third reviewer (LRP) made a final decision in case of persistent disagreement.

### Risk of individual bias of the studies

The Joanna Briggs Institute (JBI) Critical Appraisal Tools for use in JBI Systematic Reviews for randomized controlled trials (36) were utilized to assess the risk of bias and individual quality of the selected studies. Two reviewers (PUJS and DMS) independently judged each domain regarding their potential risk of bias, as recommended by the PRISMA statement (32). The percentage of “yes” answers to the questions on the assessment tool used in each study was rated as follows: the risk of bias was **high**, **moderate**, or **low** when the study obtained 49%, 50%-68%, or more than 69% “yes” answers, respectively.

### Summary measures and synthesis of results (meta-analysis)

The statistical analyses included eligible studies that provided sufficient data to calculate the relative risk (RR) of VAP in patients who received 0.12% CHX combined with toothbrushing compared with those who received 0.12% CHX alone. A meta-analysis using a random-effects model estimated the combined RRs. Three measures of heterogeneity were estimated: the τ^2^ statistic is related to the between-study variance, I^2^ reflects the percentage of variability caused by heterogeneity excluding sampling error, and H^2^ indicates the between-study level of heterogeneity (H^2^=1 indicating homogeneity). The statistical significance level was 5%.

### Certainty of evidence

The Grading of Recommendation, Assessment, Development, and Evaluation (GRADE) tool with GRADE Pro GDT software (http://gdt.guidelinedevelopment.org) (37) was used to assess the certainty of evidence and strength of recommendation. The basis for this assessment was the study design, risk of bias, inconsistency, indirect evidence, imprecision, and publication bias. The level of certainty among the identified evidence was characterized as high, moderate, low, or very low (37).

## RESULTS

### Study selection

The first phase of the study selection revealed a total of 2,337 works. The “gray literature” did not disclose any studies related to the objective of this systematic review. After discarding duplicates, 1,071 papers remained for title and abstract screening. After a detailed analysis, only four studies were eligible for full-text review. The references from these studies did not reveal additional articles of interest; after reading the full text, the qualitative analysis did not exclude any of the four selected studies. Figure 1 presents the process of search, identification, inclusion, and exclusion of studies.

### Study characteristics of eligible studies

The studies were published between 2009 and 2017, and the patients from the United States (16), Spain (2,38), and Brazil (39) were included in these studies. Overall, 988 patients were included in the analysis. Sources of information on the demographic and clinical characteristics of the population are presented in Table 2. The Ethics Committee of their respective institution or hospital approved all selected studies, which also provided informed consent before patient recruitment. None of the studies followed the Consolidated Standards of Reporting Trials statement. Half of the selected studies reported calibration among nurses (38) and dentists (39) who performed oral hygiene procedures. Two studies (38,39) presented the registration number of randomized controlled clinical trials.

### Risk of individual bias

Table 3 shows the risk of bias and individual quality of the selected studies (2,16,38,39). One study (2) did not provide details regarding the randomization procedure. None of study was blinded because the participants were admitted to the intensive care units under invasive mechanical ventilation in an unconscious state. Questions 5 and 6 were answered as “unclear” in three studies (2,16,38) because it was not clear if those applying the treatment were aware of the allocation of the participants. The answer to question 7 was considered “no” in two studies (16,39) because the groups were not treated identically according to the intervention of interest.

### Outcomes of each study

Three studies reported the VAP incidence rate and average number of days of ventilation (2,38,39). None of the studies found a significant difference between CHX+toothbrushing and CHX alone in preventing VAP, except one study (16) that compared groups with inadequate answers to the present question. Table 4 shows other outcomes common to two or more studies. All studies (2,16,38,39) reported positive results on microbiological tests for VAP identification. Moreover, none of the studies reported mortality rates.

### Synthesis of meta-analysis

**Figure 2:**
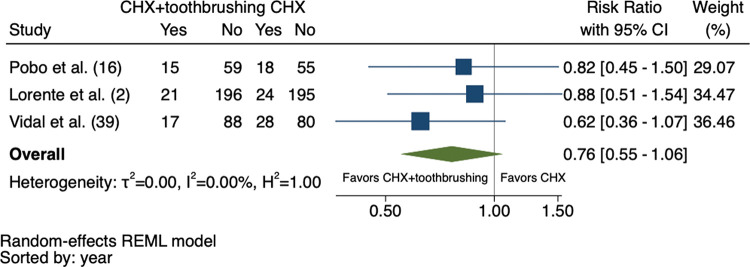
Forest plot comparing the CHX 0.12% + toothbrushing and CHX 0.12% alone groups.

The meta-analysis did not include one of the four eligible studies in the systematic review due to the lack of comparison between the intervention and control groups (16). As shown in Figure 2, there was a 24% reduction in the RR of VAP in patients who underwent CHX + toothbrushing, although this effect was not considered significant (RR: 0.76; 95% confidence interval: 0.55-1.06). The heterogeneity between the studies was low (I^2^=0%, τ^2^=0%, H^2^=1.00).

### Certainty of evidence collection

The certainty of evidence from the outcome evaluated by the GRADE approach (37) was assessed as “moderate,” which means that the true effect is likely to be close to the estimated the effect, although there is a possibility that it is substantially different. Table 5 shows more details regarding the evaluation of each GRADE tool domain.

## DISCUSSION

This systematic review of the literature compared the performance of 0.12% CHX alone and 0.12% CHX with toothbrushing in the prevention of VAP in adults requiring mechanical ventilation in intensive care units. The results of the meta-analysis revealed a non-significant 24% reduction in the frequency of VAP in the CHX + toothbrushing group as opposed to the group that exclusively used CHX. This reduction in the incidence of VAP suggests the protective effect of toothbrushing associated with CHX, but must be interpreted with caution due to the lack of significant results.

The oral cavity microbiota is highly diverse and dynamic, mainly due to the wide variety of microbial habitats in the mouth and changes that can arise in these environments due to the adjustment in diet, salivary flow, and oral hygiene interventions (40-44). The oral cavity directly connects to the lower airways; therefore, there is an alleged association between oral microbiology and respiratory infections (45). Carrilho-Neto et al. (46) showed a reduction in oral hygiene in most hospitalized patients, and reported a positive correlation between the dental plaque index and gingival inflammation index (46). In intubated patients, gingival inflammation caused by inadequate oral hygiene has also been associated with lung inflammation (46-48).

Dental plaque accumulation and colonization of microorganisms in the mouth were significantly higher from day four of intubation, conferring a higher risk of VAP (49). Sands et al. (45) revealed that in one-third of mechanically ventilated patients, dental plaque is presumed to be a reservoir of certain respiratory pathogens such as *Staphylococcus aureus* and *Pseudomonas aeruginosa* (45). In one of the eligible studies, *S. aureus* and *Haemophilus influenzae*, organisms connected with respiratory infections, were abundant in dental plaque (50,51).

In 2020, the COVID-19 pandemic scenario contributed to the need for prolonged mechanical ventilation in infected patients, since intubation is frequent in those with more severe cases, also increasing the incidence of COVID-19-related pneumonia (52). The clinical presentation of COVID-19 pneumonia is homogeneous, which greatly overlaps with that of VAP. This situation hinders the use of empiric antibiotics due to the increased risk of multi-drug resistance (6). SARS-CoV-2 infection induces increased cytokine production, causing immune dysregulation and the development of hyperinflammation and defects in lymphoid function (9,10). In addition, the virus has the ability to infect hair cells in the alveoli, decreasing the airway clearance capacity and evolving to respiratory distress syndrome (53). This complication observed in patients hospitalized with COVID-19 is managed by mechanical ventilation (54). There are still serious risks of bacterial infections related to VAP in patients with COVID-19. Coinfection can worsen the clinical picture and increase the mortality of patients with COVID-19, as well as prolong and increase hospitalization costs. When VAP cannot be prevented in patients with COVID-19, this infection must be identified early to increase the chances of successful treatment (55).

CHX has been recognized as the gold standard in oral hygiene care and maintenance for over 20 years (56,57). It is an effective ally in the control of plaque and treatment of gum disease, when associated with brushing (57,58), in addition to diseases such as alveolar osteitis and bacteremia after tooth extractions (59). Its use was also considered safe in patients who received implants because it has excellent resistance to titanium corrosion (60).

CHX is a cationic biguanide with lipophilic groups that can bind to bacterial cell walls and alter their osmotic balance (13,14). This effect inhibits bacterial growth and can even prevent the death of patients; the mechanism of action depends on the concentration of the substance (15). In addition to CHX, toothbrushing has shown promising effects on VAP (61,62). Disorganization of plaque or biofilm adherent to the dental surface can be performed mechanically and chemically (63). Brushing assists in the removal of biofilm through the brush bristles, as mechanical contact can break plaque that is adherent to the tooth surface (64,65). Disruption of dental plaque through toothbrushing facilitates the action of CHX on residual biofilms.

Meinberg et al. (17) conducted a clinical trial using CHX (2%) with and without toothbrushing and observed that 55.8% of patients developed VAP (17). All studies included in the present review showed reduced VAP incidence rates (2,38,39). This result supports the use of 0.12% CHX in VAP prevention care in mechanically ventilated patients. Additionally, the use of CHX at high concentrations presumably causes adverse effects, such as oral mucosal irritation (66) and the development of respiratory distress syndrome (RDS) due to the ingestion of CHX (67). RDS is associated with diffuse alveolar and endothelial lesions (68), which can be fatal in fragile patients (69).

Other adverse effects caused by the mechanism of action of CHX, as well as its prolonged use, include changes in taste (70) and pigmentation in the enamel, tongue, and composite resin fillings (71). In an attempt to minimize or to eradicate such effects, researchers have sought changes in the use of this molecule. Guerra et al. (71) demonstrated that the decrease in the concentration of CHX with cetylpyridinium chloride maintains a protective effect without changes in flavor perceived by the patient (71). In a pilot study by Ripari et al. (72), the efficacy of CHX mouthwash and tea tree oil was compared in the treatment of gingivitis; results suggest that tea tree oil may be advantageous in cases where patients spend little time brushing their teeth (72).

VAP increases the period of mechanical ventilation, which has been related to high patient morbidity and mortality rates, as well as increased hospital costs (69,73). In any of the eligible studies in the present systematic review, the comparison between toothbrushing combined with CHX (0.12%) and CHX (0.12%) alone did not reveal a significant reduction in the number of days of mechanical ventilation (2,38). This result may show that the hospital length of stay associated with mechanical ventilation is a risk factor that overlaps with the VAP prevention protocol. One of the eligible studies observed this relationship, in which the majority of VAP cases occurred after day four of mechanical ventilation (39).

Among the included articles, it is possible to recognize that toothbrushing alone is not superior in inhibiting VAP over 0.12% CHX alone (17,38). Manual brushing with CHX does not help prevent VAP among patients receiving intensive mechanical ventilation therapy (2). However, although not significant, the meta-analysis conducted in our study showed a 24% reduction in the incidence of VAP in the CHX (0.12%) + toothbrushing group. This result may demonstrate the protective role of brushing in preventing VAP; however, due to the lack of statistical power, this did not reach the significance level. This also corroborates the study of Yao et al. (74), who assessed the risk of VAP using toothbrushing with purified water and revealed a VAP incidence of 34% (74). Among the eligible studies, the incidence of VAP ranged from 10.3% (2) to 22.4% (38).

VAP has considerable mortality rates, although the cause of death may be associated with previous morbidity (75). The attributable mortality associated with VAP is approximately 10%, ranging from 3% to 22% (76,77). Eligible trials included in the present study did not report the VAP mortality rates, representing an important limitation of the present conclusions.

This systematic review and meta-analysis has other limitations. First, only a small number of studies were included in the review. Second, eligible studies showed lack of relevant information, such as patient mortality and overall length of stay in intensive care units. Thus, our results should be interpreted with caution, and further studies with a standardized design are warranted to examine the use of 0.12% CHX + toothbrushing in reducing the risk of VAP in patients undergoing mechanical ventilation in intensive care units. As a strength, our review had a very comprehensive search strategy, including part of the gray literature; to the best of our knowledge, this is the first meta-analysis of clinical trials to compare the CHX (0.12%) + toothbrushing and CHX (0.12%) protocols.

## CONCLUSION

Considering the limitations of this study, a standard protocol for the prevention of VAP is not recommended. Healthcare professionals should be aware of the benefits of oral hygiene in intensive care unit patients, to primarily reduce the incidence of VAP. The adoption of CHX may represent an improvement in mortality rates of patients under mechanical ventilation and, consequently, an improvement in patients’ quality of life, as well as a reduction in hospital expenses. Future research should focus on a single VAP prevention protocol using CHX+toothbrushing, including large sample sizes, aspects related to length of hospital stay, and mortality.

## AUTHOR CONTRIBUTIONS

Silva PUJ and Cardoso SV conceived the idea and played full roles in the identification, article review, data extraction, quality assessment, analysis, draft writing, and revision of the manuscript. Meneses-Santos D, Macedo DR, Blumenberg C and Paranhos LR played major roles in the analysis, manuscript draft preparation, and revision. All authors have read and approved the final version of the manuscript for publication. All authors agreed to be equally accountable for all aspects of this study.

## Figures and Tables

**Table 1:** Electronic databases and applied search strategy.

Database	Search strategy (April, 2020)
	
**PubMed** https://www.ncbi.nlm.nih.gov/pubmed	((“Pneumonia, Ventilator-Associated” OR “Pneumonia, Ventilator-Associated” OR “Ventilator Pneumonia Associated” OR “Ventilator-Associated Pneumonia”) AND (“Chlorhexidine” OR “Chlorhexidine Gluconate”))
	
**Scopus** http://www.scopus.com/	((“Pneumonia, Ventilator Associated” OR “Pneumonia, Ventilator Associated” OR “Ventilator Pneumonia Associated” OR “Ventilator-Associated Pneumonia”) AND (“Chlorhexidine” OR “Chlorhexidine Gluconate”))
	
**Embase** http://www.embase.com/	(“Pneumonia, Ventilator Associated” OR “Pneumonia, Ventilator Associated”/Exp OR “Pneumonia, Ventilator-Associated” OR “Ventilator Pneumonia Associated” OR “Ventilator-Associated Pneumonia”/Exp OR “Ventilator-Associated Pneumonia”) AND (“Chlorhexidine”/Exp OR “Chlorhexidine” OR “Chlorhexidine Gluconate”/Exp OR “Chlorhexidine Gluconate”)
	
**SciELO** www.scielo.org	“Pneumonia Ventilator Associated” AND “Chlorhexidine”
	
**Web of Science** http://apps.webofknowledge.com/	((“Pneumonia, Ventilator Associated” OR “Pneumonia, Ventilator Associated” OR “Ventilator Pneumonia Associated” OR “Ventilator-Associated Pneumonia”) AND (“Chlorhexidine” OR “Chlorhexidine Gluconate”))
	
**LILACS** lilacs.bvsalud.org	tw:(tw:(“Pneumonia Ventilator Associated” AND “Chlorhexidine”) AND (db:(“LILACS”)))
	
**Cochrane** https://www.cochranelibrary.com/	((“Pneumonia, Ventilator Associated” OR “Pneumonia, Ventilator Associated” OR “Ventilator Pneumonia Associated” OR “Ventilator-Associated Pneumonia”) AND (“Chlorhexidine” OR “Chlorhexidine Gluconate”))
**LIVIVO** https://www.livivo.de/app	((“Pneumonia, Ventilator Associated” OR “Pneumonia, Ventilator Associated” OR “Ventilator Pneumonia Associated” OR “Ventilator-Associated Pneumonia”) AND (“Chlorhexidine” OR “Chlorhexidine Gluconate”))
	
**OpenThesis** http://www.openthesis.org/	((“Pneumonia, Ventilator Associated” OR “Pneumonia, Ventilator Associated” OR “Ventilator Pneumonia Associated” OR “Ventilator-Associated Pneumonia”) AND (“Chlorhexidine” OR “Chlorhexidine Gluconate”))
	
**Open Access Thesis and Dissertations** https://oatd.org/	((“Pneumonia, Ventilator Associated” OR “Pneumonia, Ventilator Associated” OR “Ventilator Pneumonia Associated” OR “Ventilator-Associated Pneumonia”) AND (“Chlorhexidine” OR “Chlorhexidine Gluconate”))

**Table 2:** Summary of the main characteristics of the eligible studies (all were randomized clinical trials with previous ethical clearance and application of informed consent, with patients receiving mechanical ventilation for more than 48 hours without pneumonia at baseline).

Author (year)	Country	Participants	Groups	Sex	Age: Mean (SD)	APACHE, [type]: Mean (SD) [26,27]
Munro et al. (16)	United States	537 patients				[APACHE III]
			Intervention 1: Toothbrushing (three times daily)	M: 28 F: 21	47.9 (17.5)	76.4 (23.3)
			Intervention 2: Toothbrushing (three times daily) + 0.12% CHX (twice daily)	M: 28 F: 20	47.3 (18.8)	76.2 (25.5)
			Control 1: 0.12% CHX/swab (twice daily)	M: 26 F: 18	46.1 (18.2)	80.4 (28.7)
			Control 2: usual care (NR)	M: 37 F: 14	46.8 (16.4)	76.2 (3.3)
Pobo et al. (38)	Spain	147 patients	Intervention: Standard care + toothbrushing (three times daily)	M: 49 F: 25	55.3 (17.9)	[APACHE II] 18.8 (7.1)
			Control: Standard care (gauze containing 20 mL of 0.12% CHX applied to teeth, tongue, and the mucosal surface + 10 mL of 0.12% CHX digluconate was injected into the oral cavity (three times daily)	M: 46 F: 27	52.6 (17.2)	18.7 (7.3)
Lorente et al. (2)	Spain	436 patients	Intervention: 0.12% CHX‐impregnated gauze + toothbrushing of the teeth with 0.12% CHX (three times daily)	M: 146 F: 71	61 (15.6)	[APACHE II] 17.88 (8.84)
			Control: 0.12% CHX‐impregnated gauze and oral cavity injection only (three times daily)	M: 145 F: 74	60.4 (16.6)	19.16 (9.88)
Vidal et al. (39)	Brazil	213 patients	Intervention: toothbrushing + 0.12% CHX (twice daily)	M: 51 F: 54	59.4 (14.5)	[APACHE II] 21.9 (7.5)
			Control: swab + 0.12% CHX (twice daily)	M: 54 F: 54	63.2 (14.5)	22.2 (7.7)

SD, standard deviation; APACHE, Acute Physiology and Chronic Health Evaluation; M, male; F, female; CHX, chlorhexidine.

**Table 3:** Risk of bias assessed by the Joanna Briggs Institute Critical Appraisal Tools for use in JBI Systematic Reviews for randomized clinical trial studies.

Authors	Q.1	Q.2	Q.3	Q.4	Q.5	Q.6	Q.7	Q.8	Q.9	Q.10	Q.11	Q.12	Q.13	% yes/risk
Munro et al. (16)	√	√	√	N/A	U	U	--	√	√	√	√	√	√	69.2%/low risk of bias
Pobo et al. (38)	√	√	√	N/A	U	U	--	√	√	√	√	√	√	69.2%/low risk of bias
Lorente et al. (2)	√	U	√	N/A	U	U	√	√	√	√	√	√	√	69.2%/low risk of bias
Vidal et al. (39)	√	√	√	N/A	√	√	√	√	√	√	√	√	√	92.3%/low risk of bias

Q.1: Was true randomization used for the assignment of participants to treatment groups? Q.2: Was allocation to treatment groups concealed? Q.3: Were treatment groups similar at baseline? Q.4: Were participants blinded to the treatment assignment? Q.5: Were those who delivered treatment blinded to the treatment assignment? Q.6: Were outcome assessors blinded to the treatment assignment? Q.7: Were the treatment groups treated identically other than the intervention of interest? Q.8: Was the follow-up completed, and if not, were the differences between groups in terms of their follow-up adequately described and analyzed? Q.9: Were participants analyzed in the groups to which they were randomized? Q.10: Were outcomes measured in the same way for the treatment groups? Q.11: Were outcomes measured in a reliable way? Q.12: Was appropriate statistical analysis used? Q.13: Was the trial design appropriate, and any deviations from the standard RCT design (individual randomization, parallel groups) accounted for in the conduct and analysis of the trial? √, yes; --, no; U - uncertain; N/A - not applicable.

**Table 4:** Summary of the outcomes of the eligible studies.

Author	VAP incidence	Days ventilated, Mean (SD)	Mortality (VAP)	Microbiology
Munro et al. (16)	IG1: NR/49 IG2: NR/48 CG1: NR/44 CG2: NR/51	NR	NR	Yes
Pobo et al. (38)	IG:15/74 CG:18/73	8.9 (5.8) 9.8 (6.1)	NR	Yes
Lorente et al. (2)	IG: 21/217 CG: 24/219	9.18 (14.13) 9.93 (15.39)	NR	Yes
Vidal et al. (39)	IG:17/105 CG: 28/108	8.7 (5.0) 11.1 (7.6)	NR	Yes

IG, intervention group; CG, control group.

**Table 5:** Grading of the Recommendations Assessment, Development, and Evaluation (GRADE) Summary of Findings Table for the Outcomes of the Systematic Review and Meta-Analysis.

Certainty assessment						No. of patients		Effect		Certainty
No. of studies	Risk of bias	Inconsistency	Indirectness	Imprecision	Other considerations	Experimental group	Control group	Relative (95% CI)	Absolute (95% CI)	
Is toothbrushing combined with the use of 0.12% CHX in patients undergoing mechanical ventilation more effective for preventing VAP than using CHX alone?										
3 RCTs (796 patients)	Not serious^a^	Not serious^b^	Not serious^c^	Serious^d^	None	53/396 (13.4%)	70/400 (17.5%)	RR 0.76 (0.55 to 1.06)	42 less per 1.000 (from 79 less para 11 more)	⊕⊕⊕○ Moderate

CI, confidence interval; RR, risk ratio.

a All eligible studies had a low risk of bias.

b Low heterogeneity (I^2^=0%) and overlapping confidence intervals.

c Evidence stems from studies with the population suitable for PICO.

d Confidence interval suggests no benefit in one extreme and benefit important to patients in other - rated down by one level.

**GRADE Working Group grades of evidence**.

**High certainty:** We are very confident that the true effect lies close to that of the estimated effect.

**Moderate certainty:** We are moderately confident in the effect estimate: The true effect is likely to be close to the estimated effect, although there is a possibility that it is substantially different.

**Low certainty:** Our confidence in the effect estimate is limited: The true effect may be substantially different from the estimated effect.

**Very low certainty:** We have very little confidence in the effect estimate: The true effect is likely to be substantially different from the estimated effect.
